# Case report: Metastatic melanoma derived from a somatic-type malignant transformation of a mediastinal teratoma treated with immune checkpoint inhibitors

**DOI:** 10.3389/fonc.2024.1417776

**Published:** 2024-11-13

**Authors:** Roberto Rosenfeld, Silvia Riondino, Giusy Parisi, Giovanni Maria Iannantuono, Rexhina Ajdhoni, Francesco Torino, Sabrina Mariotti, Mario Roselli

**Affiliations:** ^1^ Medical Oncology Unit, Department of Systems Medicine, University Tor Vergata, Rome, Italy; ^2^ Phase 1 Unit, Fondazione Policlinico Universitario A. Gemelli, Istituto di Ricovero e Cura a Carattere Scientifico (IRCCS), Rome, Italy

**Keywords:** teratoma, germ cell tumors, somatic-type malignancy, immune checkpoint inhibitors, case report

## Abstract

The treatment of patients affected by a teratoma with somatic-type malignancy (STM) is challenging, since they are characterized by a poor prognosis, due to chemoresistance to standard cisplatin-based regimens. Only five more case reports were described for melanomatous STM and for which there are no data available for efficacy evidences of immune checkpoint inhibitors in this setting. Here we report the case of a patient with an initial diagnosis of mediastinal pure seminoma at the first biopsy. After four cycles of a standard cisplatin-based regimen and a partial response, a radical surgery was performed, revealing a mediastinal teratoma with triple STM component (melanoma, leiomyoarcoma and primitive neuroectodermal tumor). However, during post-surgical follow-up, he developed distant metastases from the melanomatous component and a first-line treatment with immune checkpoint inhibitors (ICI) was started.

## 1 Introduction

Teratomas are defined as germ cell tumors (GCTs) consisting of tissues derived from more than one primitive germ cell layers. They can be distinguished in prepubertal- or postpubertal-type based on the presence of germ cell neoplasia *in situ*. In addition, the WHO 2016 Classification of Tumors of the Urinary System and Male Genital Organs recognizes a specific entity defined as “teratoma with somatic type malignancy” (1).

The phenomenon of a somatic-type malignancy (STM) in teratomas refers the occurrence of a secondary malignant component in the context of the tumor. It is a rare phenomenon and it has been described under a variety of names, including “secondary malignancy” and teratoma with “malignant transformation”. The main criterion for diagnosing a secondary malignancy from teratoma is represented by the overgrowth of a particular element, to the extent that others are excluded (a low-power magnification or field, 5 mm in diameter) (2). Besides, multiple somatic malignant components can be contemporary found in the same teratoma, despite this represents an even rarer event (3).

GCTs with STM represent a group of neoplasms made of various histological subtypes with different clinical and prognostic characteristics. Gonads are the most common primary site of GCTs, despite a small proportion of tumors arise in extragonadal sites presenting as retroperitoneal, mediastinal or pineal masses, in order of descending frequency. Mediastinal GCTs account for 7-12% of primary mediastinal malignancies and are frequently diagnosed in children or young men in their 3^rd^ to 4^th^ decade (4). Among the different histotypes of mediastinal GCTs, teratomas account for approximately 8% (4). STM may assume various histologies including sarcoma (commonly embryonal rhabdomyosarcomaan, less often leiomyosarcoma or angiosarcoma), primitive neuroectodermal tumor (PNET), carcinoma, glial or meningeal neoplasms, hematological neoplasms, and nephroblastoma-like tumors (2, 3, 5–12). Regarding the occurrence of a secondary malignant component attributable to melanoma, only four case reports are available in the literature (13–16).

The treatment of patients affected by a teratoma with STM is challenging. They are characterized by a poor prognosis and the radical surgery represents the milestone of the therapy. Despite a higher development of chemoresistance to standard cisplatin-based regimens in comparison to traditional GCT (11), several reports described improved outcomes in patients who were treated with chemotherapeutic regimens tailored to the somatic component (8). In contrast, to our knowledge no data are available for immune checkpoint inhibitors in this setting.

Here we report of a patient diagnosed with a mediastinal teratoma with multiple STM (melanoma, leiomyosarcoma, and PNET) treated with neoadjuvant standard cisplatin-based regimen, who underwent radical surgery. After three months of follow-up the patient developed distant metastases from the melanomatous component and, thus, started a first-line treatment with immune checkpoint inhibitors (ICI). This case report was described according to the CARE guidelines.

## 2 Case description

**Figure 1:**
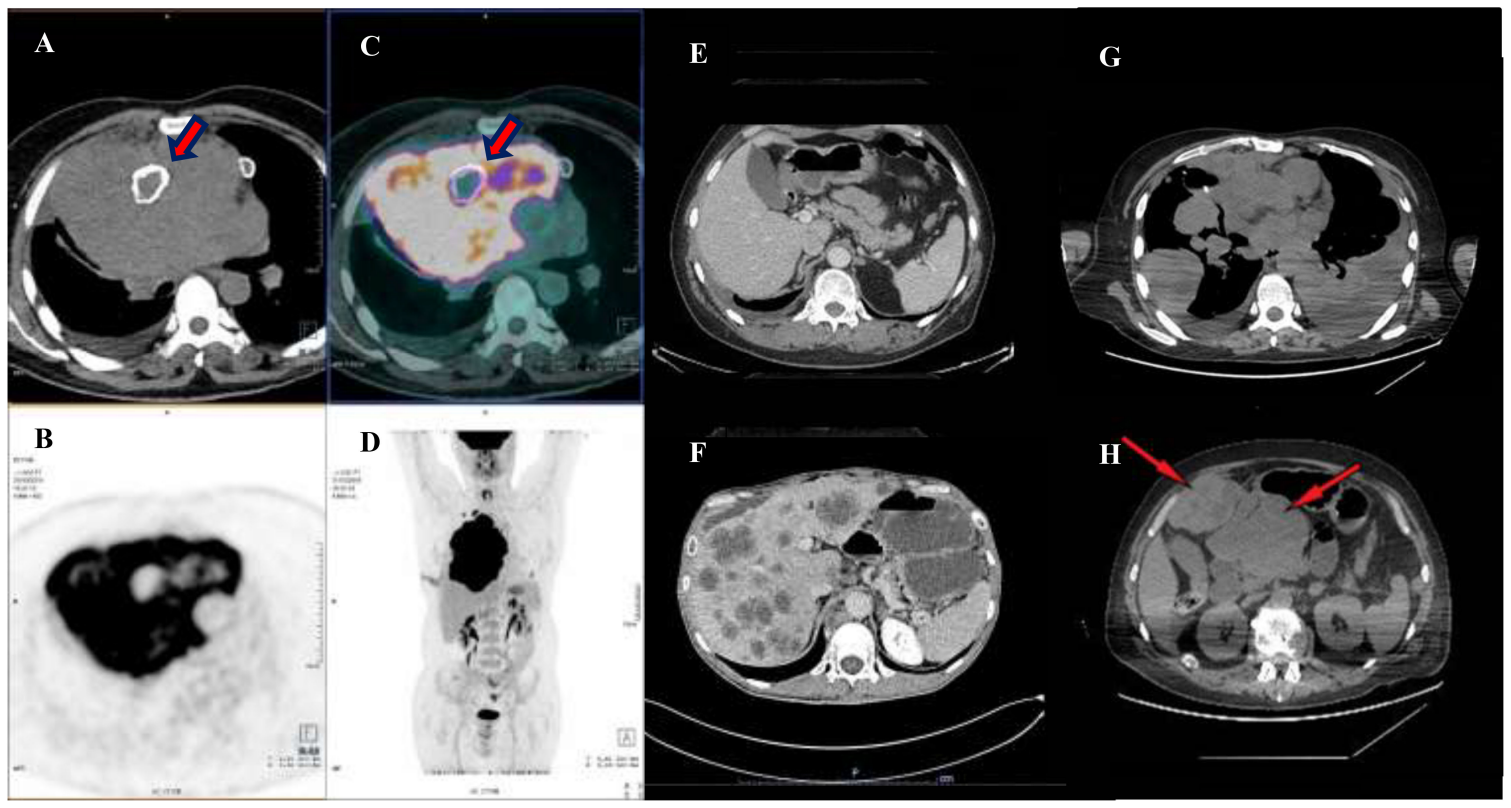
Preoperative and pre-chemotherapy PET-CT scan. **(A–D)** shows a PET-CT scan performed in October 2019, evidencing a bulky expansive lesion occupying the large part of mediastinum and dislocating the right lung peripherally. Note **thick red arrows** pointing to a calcified necrotic center, englobed in the neoplatic mass. Thereafter, an induction chemotherapy was performed with the aim of reducing the mass and leading the patient to the surgical excision. Image in **(E)** shows a post-surgical CT scan performed in April 2020, during outpatient clinic follow up, note that the abdomen window was free from disease. However, an early relapse occurred in August 2020 with development of hepatic metastases **(F)**. A first line of therapy SMT-oriented, with a sequential use of Nivolumab and Ipilimumab was used. However, after four months of therapy, a restaging with a CT scan was performed highlighting a progressive disease. The disease spread in the lung bilaterally **(G)** and in the abdomen **(H)** with a disease extension of the II and III hepatic segments over the epigastrium (thin red arrows).

In September 2019, a non-smoker 61-year-old man, without relevant medical history except for mild hypertension, referred to his general practitioner complaining of a dry cough for the past two weeks. A chest X-ray showed a radio-opacity attributable to a mediastinal mass. A 18F-fluorodeoxyglucose positron emission tomography with integrated computed tomography (FDG-PET/CT) scan showed a 14x15x18 cm right anterior mediastinal solid mass associated with an inhomogeneous contrast-enhanced internal necrotic area, superior vena cava compression, left brachiocephalic vein compression and right pleural and pericardial effusion (**Figures 1A–D**).

In October 2019, a video-assisted thoracoscopic guided biopsy of the mediastinal mass was performed and histological examination of the samples revealed the presence of a seminoma (PLAP+, cKit+, PanCK+/-, S100, CK 5/6-, CD30-, TTF1-, p40-, CD20-, CD3-, LCA-). In addition, thoracentesis and pericardiocentesis were carried out, but cytological analysis of pleural and pericardial effusions did not reveal cancer cells. Testicular ultrasonography was negative. Serum β-human chorionic gonadotropin (β-HCG), alpha-fetoprotein (αFP) and lactate dehydrogenase (LDH) levels were 13.94 mIU/ml (normal range 0 - 5 mIU/ml), 7301 IU/ml (normal range 0 – 6.72 IU/ml) and 6835 U/L (normal range 125 – 220 U/L), respectively.

Based on those data, a locally-advanced mediastinal seminoma was diagnosed. The patient was not candidate to an upfront surgical removal of the mediastinal mass and, thus, in November 2019 he underwent cytotoxic chemotherapy with the combination BEP (bleomycin 30 units IV weekly on days 2,9 and 16; etoposide 100 mg/m2 on days 1-5; cisplatin 20 mg/m2 on days 1-5, cycles repeated every 21 days) for four cycles. At the end of treatment, the patient repeated a 18F-fluorodeoxyglucose positron emission tomography with integrated computed tomography (FDG-PET/CT) scan that showed a reduction of the mediastinal mass (13 x 9 x 10 cm) with the resolution of pleuro-pericardia effusions and the mediastinal vessels’ compression (**Figures 1E, F**).

Considering the partial response, as per RECIST criteria, detected with FDG-PET/CT, the patient was re-evaluated in a multidisciplinary meeting and, shortly thereafter, in May 2020 he underwent the removal of the mediastinal mass together with a right pulmonary resection. The histological examination showed a teratoma with both immature and mature components associated with a minimal presence of seminoma. Besides, the presence of multiple malignant somatic components attributable to melanoma (SOX10+, MART1+, HMB45+, VIM+, S100+/-), leiomyosarcoma (alpha-actin+,desmin+), and PNET (CD99+, αFP+) was documented. Surgical margins were clear. A week after surgery procedure, β-HCG, αFP, and LDH levels were <1 mUI/ml, 101.4 UI/ml, and 160 U/L, respectively. Thus, the patient started the follow-up in June 2020.

In August 2020, a new CT scan revealed pulmonary, hepatic, and bone metastases. In September 2020, a CT-guided biopsy of pulmonary and bone metastases was performed. Histopathology showed the presence of melanoma cells (HMB45+, SOX10+, desmin-, panCK-) with a negative immunohistochemistry for V600E BRAF mutation and positive for c-Kit. A dermatological physical examination excluded any primary cutaneous or mucosal melanoma. Therefore, the patient started a first-line treatment for advanced melanoma with the PD-1 ICI, Nivolumab in October 2020, without associating cytotoxic T lymphocyte antigen-4 (CTLA-4) inhibitor, Ipilimumab, according to the Italian Drug Regulatory Agency (AIFA) directives.

The patient referred to a tertiary Center to continue the ongoing treatment with ICI. After 6 cycles with Nivolumab, the instrumental re-assessment found a widely extended, progressive disease, and a therapy with anti-CTLA-4 Ipilimumab was promptly started. At the same time, the mutational analysis of c-Kit was requested, revealing the D816Y mutation on exon 17 and disclosing Imatinib mesylate, for a hypothetical fourth metastatic line of treatment. Unluckily, in the intervening time, the patient’s general conditions worsened and he referred to the Emergency Room of our hospital with a diagnosis of a strangulated hernia. Eventually, he died for septic shock. **Table 1** reports a timeline with relevant data from the episode of care.

**Table 1:** Timeline of dates for the relevant episode of care.

Treatment stage	Dates
VAT-guided biopsy of the mediastinal mass	October 2019
Basal PET-CT scan	October 2019
Chemotherapy with BEP protocol	November 2019
Surgical removal of mediastinal mass	May 2020
Follow-up	June 2020
Radiological assessment of progressive disease	August 2020
Pulmonary and bone biopsies	September 2020
First-line treatment with Nivolumab	October 2020
Progressive Disease	December 2020
Exitus	December 2020

BEP, bleomycin etoposide and cisplatin; VAT, video-assisted thoracoscopy.

## 3 Discussion

The occurrence of a triple histology of STMs is a rare event hardly described in case series and retrospective cohort analysis (3, 12, 17, 18) and the development of a melanomatous component is a unique feature described only in other 4 case reports in literature. Our case report showed both this characteristics, moreover an STM-oriented therapy was chosen and Nivolumab was administered for the first time in literature in a male GCT with STM.

The phenomenon of STM in teratomas refers to the occurrence of a secondary malignant component that may be found in the late stages of various GCT histotypes, but in large part of cases is associated with teratomas (2). Although rare, several case series or retrospective studies on this pathology available in the literature (3, 5–12) (**Table 2**), described that the commonest histologies were sarcoma, PNET and carcinoma (3, 12, 18, 19), whereas no mention was made of the occurrence of a somatic malignant component attributable to melanoma. Indeed, only other four reports were found querying Pubmed database, assessing this feature as an even rarer event (3, 12).

**Table 2:** Case series or retrospective studies on the phenomenon of STM in teratomas.

Authors	N. teratomas /N. of total cases	Patients median age	Stages at diagnosis (relative frequency)	Primary site (Abs freq./Rel. freq.)	Histology frequencies (absolute frequency)	Possible prognostic factors
		**(IQR** **range)**				
					1. Rabdomiosarcoma (6)	
					2. Other sarcomas (6)	
				1. Testis: 7	3. Nefroblastoma (2)	Worse Prognosis:
Ulbright et al. (1984)	10/11 (91%)	30 (13)	Not described	2. Mediastinum: 3 3. Retroperitoneum: 1	4. Neuroblastoma (1) 5. Malignant giant cell tumor (1)	Rhabdomyosarcoma Histologies
					6 Adenosquamous Ca (1)	
					7. Gliobastoma (1)	
					1. Carcinoma (6)	
Comiter et al. (1998)	21/21 (100%)	Not known	Stage I: 1 (5) Stage II-III: 20 (95)	1. Testis: 18 2. Mediastinum: 2 3. Retroperitoneum: 1	2. Rhabdomyosarcoma (4) 3. Other sarcomas (6) 4. Malignant peripheral nerve sheath tumor (3) 5. PNET (2)	Worse prognosis: a) Mediastinal primitivity, b) PNET and Rhabdomyosarcoma histologies
Motzer et al. (1998)	26/46 (57%)	27 (16-56)	Stage I: 4 (9) Stage II: 20 (43) Stage III: 22 (48)	1. Testis: 36 2. Mediastinum: 9 3. Retroperitoneum: 1	1. Rhabdomyosarcoma (16) 2. Sarcoma NOS (11) 3. Adenocarcinomas (10) 4. PNET (7) 5. Leukemia/lymphoma (4)	Worse Prognosis: a) Incomplete surgery b) Late stages c) Mediastinum as primary site vs Testis
					1. Rabdomiosarcoma (4)	
Donadio et al. (2003)	Not known n =10	30 (19-55)	Stage I: 2 (20) Stage II: 2 (20) Stage III: 6 (60)	1. Testis: 6 2. Mediastinum: 4	2. Adenocarcinoma (2) 3. Leukemia (2) 3. PNET (1)	Better prognosis: SMT-oriented CT
					4. Anaplastic SCT (1)	
Malagòn et al. (2007)	42/46 (91%)	26.5 (22-30)	Stage I: 4 (9) Stage II: 6 (13) Stage III: 28 (61) Unknown: 7 (13)	1. Testis: 18 2. Mediastinum: 23 3. Retroperitoneum: 1 4. Ovary: 3	a) Rabdomiosarcoma (29) b) Angiosacroma (6) c) Leiomiasrcoma (4) d) Myxoid liposarcoma (1) e) MPNST (1) g) Epithelioid hemangio- endothelioma (1)	Worse Prognosis: a) Sarcomatous SMT b) Primary site
El Mesbahi et al. (2007)	8/14 (57%)	29.5 (19-45)	Stage I: 3 (21) Stage II: 2 (14) Stage III: 9 (65)	1. Testis: 9 2. Mediastinum: 4 3. Retroperitoneum: 1	1. Sarcoma (10) 2. Adenocarcinoma (3) 3. Broncoalveolar Ca (1)	Worse Prognosis: a) Incomplete surgery b) SMT presence

Ca, carcinoma; CHT, chemotherapy; CT, computed tomography; GCT, germ cell tumors; MPNST, Malignant Peripheral Nerve Sheath Tumors; NOS, not otherwise specified; PNET, primitive neuroectodermal tumor; RPLND, Retroperitoneal lymph node dissection; SMT, somatic-type malignancy.

There are two main debated theories about the origin of a somatic malignant component and the old distinction between mature and immature teratoma could still be useful. According to a first hypothesis, a somatic-type malignancy could derive from the differentiation of a totipotent tumoral cell, a common progenitor, which could develop two different parallel lines, the teratoma and the STM, phylogenetically related (20, 21). Conversely, a second hypothesis points to a malignant transformation of an existing teratomatous component (5, 22) through a process of de-differentiation (3) (**Figure 1**). However, the old hypothesis that the GCT-oriented therapy could induce a differentiation from the teratomatous component seems to be only apparent as suggested by some Authors (5, 23). Teratomas with STM represent a therapeutic challenge. They are associated with poorer cancer specific survival characterized by development of chemoresistance to standard cisplatin-based regimens and a strong resilience to radiotherapy, in comparison to traditional GCTs (12, 19). In this direction, prognostic factors used for GCT lose their ability in the presence of secondary malignant components. Classically, the therapeutic options have been divided into either GCT- or STM-oriented therapy. The former is older and relies on chemotherapy schemes based on the treatment of germinal lines with contrasting results (7, 8, 10, 12). The latter has promising premises by targeting therapy to the STM component; indeed, several reports have described improved patient outcomes in those presenting with single histology a STM when treatment regimens were tailored to the somatic component (8, 12).

Concerning the detection of a malignant somatic component attributable to melanoma in mediastinal teratomas, only four case reports are available in the literature (**Table 3**). The first case report, published by McNab et al. in 2012, described a 32 years-old patient affected by a malignant melanoma arising from respiratory epithelium in a mediastinal malignant teratomatous GCT. Thirteen months after the initial diagnosis, a CT scan revealed multiple hepatic lesions attributable to distant metastases from melanoma. The Authors hypothesized that melanomatous somatic-type malignancy occurred in the teratomatous bronchial mucosa, along with other neuroendocrine cell types, could be explained by the “dispersed neuroendocrine system” (DNES) theory. This suggested a derivation from neural crest cells that, in turn, arose out of the neuroectodermal layer of the developing embryo. Notably, in accordance with McNab theory, in our case we observed the association with PNET and melanoma as somatic- type malignant transformation, suggesting either a common origin or the evolution of the second tumor from the former one (13). The second case report published by Mustafa et al. in 2016, described a 21 years-old man who was diagnosed with an immature mediastinal teratoma with multiple malignant components attributable to sarcoma, carcinoma, and melanoma. Unfortunately, after the surgical removal of the tumor, the patient developed multiple liver and bone metastases from melanoma, which, despite the beginning of a first-line treatment with Temozolomide, brought the patient to exitus a few months later (14). Analogously, our patient was affected by multiple co-existing somatic malignancies with the main aggressive component being a melanoma. Of note, Mustafa et al. observed the co- existence of immature neuroectodermal cells with melanoma, whereas we found an association between PNET and melanoma and this could, intriguingly, reinforce McNab’s theory. The third case report published by Nozaki et al. in 2018, described a 14-year-old affected by a malignant teratoma with areas of yolk sac tumor, who developed distant metastases attributable to melanoma after surgical removal of the mediastinal mass. A retrospective revision of the histopathological slide detected a melanomatous component (15). Lastly, Lee et al. published in 2020 the story of a 32-year-old patient diagnosed with a mediastinal teratoma with a somatic malignant component attributable to melanoma, without evidence of distant metastases. Although the patient was disease-free at the time of publication, we don’t know if there has been any evolution of the patient’s health status nor of possible therapies (16). Eventually, our case report differed to all those previously published for the evident older age (13–16), furthermore similarly only to the Mustafa’s case report and confirmed by several case series (3, 12, 19), the finding of a triple somatic malignant component is a very rare event, as well as a poor prognostic factor (12, 19). Moreover, our clinical case in agreement with the previous ones highlighted that STM and the development of metastases from the melanomatous component determined a poorer prognosis for the patients. Finally, a common development by neural crest and/or PNET might suggest different mutational drivers than classical melanoma oncogenesis, in addition to the abundant evidence that PNET acts as a worse independent prognostic factor (12). From the available evidences, we expected a poor prognosis for which an effective and innovative therapy was needed, leading our choice toward a therapy oriented to the somatic malignant component. Nowadays ICIs have changed the landscape of the treatment of advanced melanoma and the administration of these drugs in primary metastatic melanoma is recommended. To our best knowledge, we proposed the first case of teratoma with multiple distant metastases derived from the melanomatous somatic component treated with immune checkpoint inhibitors. In literature, two similar cases were described, which used immunotherapy as first line treatment. The first one, described a 63-year-old woman affected by an ovarian teratoma, who developed distant metastases from a melanomatous component, after which a sequential treatment with Ipilimumab, radiotherapy, Nivolumab and Pembrolizumab was chosen. However, the benefit was poor and a slow progression until death was observed (24). The second case reported a patient affected by a mediastinal germ cell tumor with a large teratomatous component with melanocytic neuroectodermal tumor, likely deriving from thymic tissue (25). The lesion was surgically removed but the patient developed bone metastases from melanocytic neuroectodermal tumor, ten years later. Interestingly, the gene profiling performed resulted to be similar to a melanoma profile. This 39 year-old patient was treated with an off-label combination of Ipilimumab and Nivolumab and he was still alive at the time of publication (25). Our case report shared similarities with both these case reports. Similar to the latter case, a neuroectodermal tumor was found as SMT (a common feature in teratoma with SMT). Interestingly, the tumor shared a common genomic profiling with melanomas, whereas our case reported a melanoma within a context of neuroectodermal tumor suggesting a common pattern of differentiation. On the other hand, the former case report showed a poor prognosis similar to our patient, thus suggesting a scarce responsivity of melanomatous somatic malignant component to ICIs.

**Table 3:** Case reports available in the literature concerning the detection of a malignant somatic component attributable to melanoma in mediastinal teratomas.

Author (Year)	Age	Initial Symptoms	Histology of biopsy	Pre-surgery chemotherapy (regimen)	Surgery of the primary tumor	Histology of the primary tumor	Post-surgery chemotherapy (regimen)	Recurrence site(s)	Histology of the recurrence
McNab et al. (2012)	32	Chest pain	Mixed GCT with seminoma, embryonal carcinoma and teratoma	Yes (BEP followed by TIP)	Resection of the mediastinal mass	Mixed GCT with melanoma	No	Liver	Melanoma
Mustafa et al. (2016)	21	Cough, chest pain and dyspnea	Immature teratoma	Yes (BEP)	Resection of mediastinal mass along with a right bi-lobectomy	Immature teratoma with PNET, sarcoma, adenocarcinoma and melanomatous components	Yes (Temozolomide)	Bones and liver	Melanoma
Nozaki et al. (2018)	14	Cough	Necrotic tissue	Yes (cisplatin based chemotherapy)	Resection of mediastinal mass with a right pneumonectomy	Teratoma with areas of yolk sac tumor and melanoma	Yes (cisplatin based chemotherapy)	Bones and liver	Melanoma
Lee et al. (2020)	34	Chest pain	Mixed GCT with yolk sac and embryonal elements	Yes (BEP followed by VIP)	Resection of the mediastinal mass, thymectomy and resection of left upper lobe	Mature teratoma with melanoma, yolk sac and embryonal elements	No	–	–

BEP, bleomycin, etoposide and cisplatin; GCT, germ cell tumors; PNET, primitive neuroectodermal tumor; TIP, paclitaxel, ifosfamide, and cisplatin; VIP, etoposide, ifosfamide, and cisplatin.

## 4 Conclusions and perspectives

This case report describes the first patient affected by a mediastinal teratoma with multiple somatic- type malignancies and distant metastases of melanomatous SMT, who was treated with ICIs. There is large disagreement in literature on the best treatment to be administered for SMTs, whether a GCT- oriented therapy or an SMT-oriented one. In our case report after choosing an SMT-oriented therapy, based on ICI, a progressive disease was observed, confirming the poor prognosis of this disease. Still, before advocating the possible scarce response to ICIs, it is important to underline that our patient developed a very important disease burden before starting Nivolumab and it is largely known that ICIs have a slow response, determining the fatal outcome. For this reason, further researches are needed on the topic to assess definitive information on the usefulness of a therapy tailored to the somatic malignant component, especially in patients who develop distant metastases. Furthermore, the peculiar biology of this type of melanoma, derived from a GCT, should be taken in account for establishing if similar prognostic results would be comparable to classical cutaneous-primitive melanoma.

## Data Availability

The raw data supporting the conclusions of this article will be made available by the authors, without undue reservation.
